# Oxidative Stress Reduction by Midazolam Premedication during Oocyte Retrieval Procedure: Pilot Study

**DOI:** 10.3390/jcm10040855

**Published:** 2021-02-19

**Authors:** Maja Pešić, Katarina Kličan-Jaić, Marinko Vučić, Krunoslav Kuna, Andro Košec, Ana Čipak Gašparović

**Affiliations:** 1 Department of Anesthesiology, Intensive Care Medicine and Pain Management, University Hospital Center “Sestre milosrdnice”, HR-10000 Zagreb, Croatia; majapesic1980@gmail.com (M.P.); katarina.klican@gmail.com (K.K.-J.); marinko.vucic@kbcsm.hr (M.V.); 2 Department of Gynecology and Obstetrics, University Hospital Center “Sestre Milosrdnice”, HR-10000 Zagreb, Croatia; krunoslav.kuna@kbcsm.hr; 3 Department of Otorhinolaryngology and Head and Neck Surgery, University Hospital Center “Sestre Milosrdnice”, HR-10000 Zagreb, Croatia; andro.kosec@yahoo.com; 4 Division of Molecular Medicine, Ruđer Bošković Institute, HR-10000 Zagreb, Croatia

**Keywords:** oocyte retrieval, midazolam, oxidative stress

## Abstract

Infertility is one of the major medical problems nowadays. Couples who opt for In Vitro Fertilization (IVF) face a great deal of stress which certainly affects the outcome of the procedure. Therefore, we aimed to reduce the stress during the oocyte retrieval procedure by applying midazolam. Total oxidant (TOC) and antioxidant (TAC) capacities of serum, as well as glutathione (GSH) content and catalase activity, were measured in both control and midazolam groups. Follicular fluid was also tested for oxidant capacity and IL1β. Results implied that the midazolam group increased TAC at the end of the procedure. At the same time, the control group decreased GSH at the beginning of the procedure, and both groups decreased catalase activity at the end of the procedure. The results imply that stress during the procedure affects oxidative and antioxidative parameters of the patients, but did not affect the frequency of the pregnancy at the end of this pilot study. Yet, the results imply that oxidative and antioxidative mechanisms during IVF should be investigated in detail as they could affect the outcome of IVF.

## 1. Introduction

Today, infertility is recognized as a growing medical problem, affecting 9% to 15% of the world’s fertile population, with the number of affected couples in Europe reaching up to 25 million [1,2]. Numerous studies show that couples who opt for In Vitro Fertilization (IVF) face a great deal of psychological and emotional stress, which is confirmed by the fact that 30% of them withdraw from the procedure before its completion due to the psychological burden they face [3]. The IVF is a multistep procedure, and one of these steps is oocyte retrieval, which is performed by transvaginal ultrasound-guided oocyte retrieval (TUGOR) [4]. TUGOR is a relatively short procedure taking 5–15 min, which depends on the number of follicles. The procedure is performed under short intravenous general anesthesia, and is guided by transvaginal ultrasound. The follicles are detected by ultrasound and the punction needle is guided by ultrasound to aspirate the oocyte in the follicular fluid. The punction causes pain, and in addition to this pain comes the fear of the needle caused by the size of the needle alone. Taking into account the fear of not obtaining (enough) oocytes, the pain caused by the needle, and the fear of needles, it is not surprising that women experience higher levels of stress compared to other stages of the procedure, and this increased anxiety before the IVF procedure reduces the number of oocytes obtained by retrieval [5,6]. However, the exact pathophysiological mechanism by which stress affects the IVF procedure and its outcome is still unknown and is thought to involve a complex interaction of the neuroendocrine, immune, and autonomic nervous systems [6,7,8,9,10]. Due to its complexity, involving several steps during the IVF procedure, stress markers, such as norepinephrine and cortisol, were found to be elevated in women who did not remain pregnant at the end of the procedure [10]. These stress markers are elevated by psychological stress and may affect the outcome [10,11]. The association of low levels of noradrenaline and cortisol at the time of TUGOR has been demonstrated with a successful fertilization outcome [6,12]. This may be attributed to the uncertainty of the success of the retrieval procedure in terms of the number of oocytes obtained, but also to the nature of the procedure itself, which is painful and uncomfortable and performed under intravenous general anesthesia [13]. Given the short duration of the procedure, patients are administered propofol, an ultrashort-acting intravenous anesthetic, and a short-acting opioid [14]. Currently, it is not common for patients to be premedicated with anxiolytics immediately before TUGOR, although few studies suggest that anxiolytic premedication reduces stress levels and pain during the procedure [12,14]. Premedication with midazolam, which is one of the most commonly used benzodiazepines, is a good choice for short-term daily surgical procedures, because it reduces stress and tension without causing more serious disorders of consciousness [12].

Oxidative stress is a disturbed balance between oxidative and reductive processes in cell, organ and organism [15]. This oxidative imbalance is both the cause and consequence of many pathological conditions and mechanism by which therapies achieve their effects [16]. Oxidative stress significantly reduces fertility in several different ways. Numerous studies link low sperm concentration and low motility to increased oxidative stress [17]. Oxidative damage of spermatozoa has also been associated with poor fertilization, pregnancy loss, birth defects and poor embryonic development [18]. In females, oxidative stress reduces oocyte quality [19] via several pathways that may include mitochondrial dysfunction, endometriosis, polycystic ovarian syndrome or premature ovarian insufficiency [20]. Additionally, manipulation with the gametes in vitro may increase reactive oxygen species [21].

The main objective of this study was to determine whether premedication with midazolam prior to oocyte retrieval affects the oxidative stress in subjects undergoing IVF by measuring oxidative stress markers in paired samples of blood serum and follicular fluid at different time points during the procedure.

## 2. Experimental Section

### 2.1. Patients and Experimental Procedure

This research was conducted in University Hospital Center “Sestre milosrdnice”. The study was approved by the Ethics Committee of UHC “Sestre milosrdnice” (EP-2258/18-3). The pilot study is registered at DRKS – German Clinical Trials Register, as a primary registry in the WHO Registry Network (Registration ID: DRKS00021657) and the ClinicalTrials.gov, ID number: NCT0416061. The procedure was explained in detail to the patients, after which the patients signed written informed consent. The total of 60 patients who underwent IVF procedure were randomized into two groups (30 patients per group): the control group and the group that received midazolam orally before the oocyte retrieval procedure. The two groups had no significant differences in clinical parameters (described in detail in the results). Blood samples from each patient were collected on admission to the hospital, before midazolam administration (time point A), 40 ± 5 min after midazolam administration, immediately before intravenous anesthesia for oocyte retrieval, (time point B), and 2 h after the procedure (time point C). Blood was then stored as whole blood, or serum at −80 °C until analyses. Retrieval A sample of follicular fluid was also collected during the oocyte and stored at −80 °C until analyses. Blood sampling was adjusted to the pharmacokinetics of midazolam and was incorporated into the medical protocol for oocyte retrieval. By comparing the groups at the first time point (A), we controlled for the uniformity of the stress response. The second time point (B) indicated the way midazolam affected the stress response at its maximum effective concentration. The third time point (C) indicated whether there was a residual effect on the stress response in the subjects’ plasma after midazolam ceased to be active.

### 2.2. Total Oxidative Capacity

Total oxidative capacity (TOC) is a colorimetric assay based on the cascade reaction of peroxides in the sample (serum and follicular fluid) with peroxidase, which produces oxygen, which in turn oxidizes tetramethyl-benzidine (TMB Sigma-Aldrich, St. Louis, MO, USA) [22]. The last reaction results in blue colour, which turns yellow when stop solution (sulfuric acid) is added. The colour intensity is measured at 450 nm (Multiscan Ex, Thermo Scientific, Waltham, MA, USA). The TOC values are calculated from the standard curve obtained with hydrogen peroxide.

### 2.3. Total Antioxidative Capacity

Total antioxidant capacity (TAC) is measured by a similar reaction to TOC, the serum samples are incubated with hydrogen peroxide which is removed by endogenous antioxidants. This is followed by the reaction of the remaining hydrogen peroxide with peroxidase and addition of TMB [22]. The reaction is stopped by sulphuric acid solution and the intensity of the yellow solution is measured at 450 nm (Multiscan Ex, Thermo Scientific, Waltham, MA, USA). The more antioxidants present in the sample, the lower the absorbance measured. 

### 2.4. Total Glutathione Levels

GSH levels were measured in whole blood lysate by the modified method by Tietze [23]. The reaction was performed in lysate samples and reaction mix (8 mM 5,5-dithio-bis-2-nitrobenzoic acid, 0.4 Units of GSH reductase, and 0.6 mM NADPH in phosphate buffer 100 mM NaH_2_PO_4_, 5 mM EDTA pH 7.4). The total GSH content was detected as yellow product, 2-nitro-5-thiobenzoic acid, and measured on the plate reader at 405 nm (Multiscan Ex, Thermo Scientific, Waltham, MA, USA). 

### 2.5. Catalase Activity

Catalase activity was measured from the whole blood lysate according to the modified method by Goth [24]. The assay is based on the fact that catalase has one of the highest turnover numbers, and is thus the first to degrade hydrogen peroxide [25]. The assay is performed using 20 µL zo, to which 65 mM H_2_O_2_ is added to start the reaction. The reaction is left for 5 min and then stopped by adding 100 μL of 200 mM ammonium molybdate and the colour development was measured spectrophotometrically in a plate reader at 450 nm (Mlutiscan Ex, Thermo Scientific, Waltham, MA, USA). The amount of degraded H_2_O_2_ is calculated from the standard curve (0 to 75 mM H_2_O_2_). One unit of catalase activity is defined as the amount of enzyme needed for degradation of 1 μmol of H_2_O_2_/min at 25 °C. Catalase activity was expressed as units per milligram of proteins in the cell lysate (U*mg^−1^).

### 2.6. Statistical Analyses

Statistical analyses were performed using Prism GraphPad 8.0 software (GraphPad software, San Diego, CA, USA), and the methods used were Student’s *t*-test for significance between the two groups and Fisher’s exact test was used to analyze the differences between hormonal status and positive outcome of IVF. Values of *p* lower than 0.05 were considered as statistically different.

## 3. Results

### 3.1. Patients

As patients were randomly assigned to each group, the two groups were analysed for possible differences in age, hormonal status (estradiol, progesterone and testosterone, dehidroxyepiandrostendione-sulfate, thyrotropin, anti-Müllerian hormone, follicle-stimulating hormone, luteinizing hormone, and prolactin), and body mass index (BMI). The two groups showed no significant difference in any of the monitored parameters (Table 1).

### 3.2. Total Oxidant and Antioxidant Capacities

**Figure 1:**
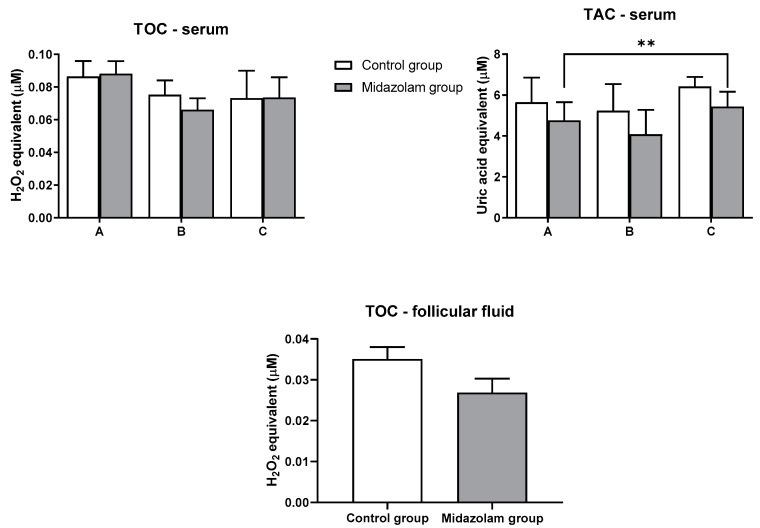
Oxidant and antioxidant capacities in blood serum and follicular fluid. Blood samples of each patient were collected at the acceptance to hospital (time point A), prior to oocyte retrieval (time point B), and 2 h after the procedure (time point C). Values represent mean ± SE; ** *p* < 0.01. TOC, total oxidant capacity; TAC, total antioxidant capacity.

Total oxidant capacity (TOC) was measured in serum and in follicular fluid at three-time points in both groups. There were no significant differences between the control group and the midazolam group at any of the observed time points in the serum and follicular fluid samples (Figure 1).

Total antioxidant capacity (TAC) was measured in serum at three time points in both groups (Figure 1), whereas for follicular fluid, TAC could not be measured due to very high TAC levels. A statistical difference was found for the third time point (C) compared to the initial time point (A) (*p* = 0.0072). However, there was no significant difference between control and midazolam groups in serum.

### 3.3. GSH Levels and Catalase Activity

**Figure 2:**
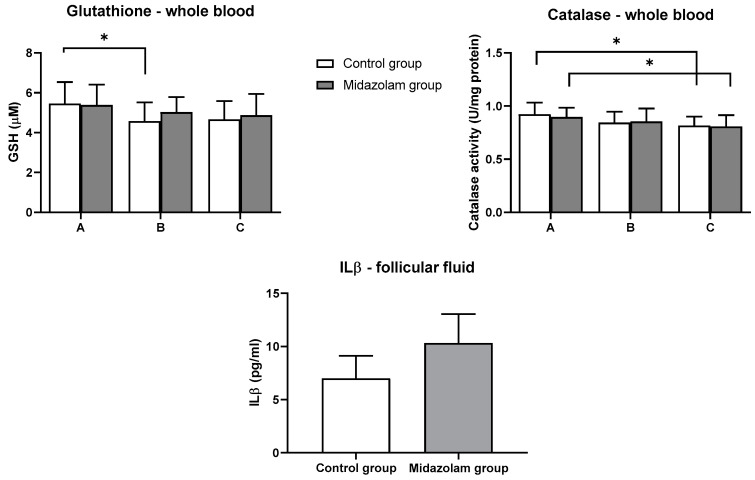
Levels of GSH and catalase activity in whole blood and levels of ILβ in the follicular fluid. Blood samples of each patient were collected at the acceptance to hospital (time point A), prior to oocyte retrieval (time point B), and 2 h after the procedure (time point C). Values represent mean ± SE; * *p* < 0.05.

Total glutathione measured in serum at three time points in both groups showed a statistically significant decrease at time point B in serum samples of the control group compared to time point A (*p* = 0.04). GSH levels at all time points in the control group were not significantly different compared to the midazolam group (Figure 2).

Catalase activity measured in whole blood samples for both groups at three time points in both groups showed a statistically significant decrease at time point C in serum samples from both groups when correlated with time point A of the respective group (*p* = 0.04). Similar to GSH, catalase activities at all time points in the control group were not significantly different from those in the midazolam group (Figure 2).

Interleukin 1β was measured in the follicular fluid of both groups, and there was no significant difference between the two groups (Figure 2).

### 3.4. Positive Outcome—Follow-Up

The follow-up rate at the end of the study period was 89%, with 8 patients not followed due to incomplete sampling or decision to leave the study. Of the total number of pregnancies (11/60), 8/30 (27%) were in the control group and 3/30 (10%) were in the midazolam group. When the distribution frequencies were analysed, the exact Fischer’s test showed no difference between control and midazolam groups. Again, we have analysed the differences in hormonal parameters between pregnancies in the control and midazolam groups and found no significant difference (Table 2).

## 4. Discussion

In order to attribute the observed differences to midazolam administration, we first eliminated possible sources of bias by ensuring that midazolam administration was the only different intervention between the groups studied. The random selection revealed no statistically significant differences between the two groups in factors shown to influence the outcome of the procedure. We hypothesized that the anxiolytic and sedative effects of midazolam would reduce systemic stress during oocyte retrieval in IVF. A reduction in systemic stress would then produce a measurable difference in markers of oxidative stress in patients’ serum and follicular fluid, and thus have a potential beneficial effect on the outcome of IVF. To date, no studies have investigated the effect of premedication in IVF procedures, making a comparative analysis of our results difficult.

Oxidative stress is a shift of equilibrium in oxidation-reduction processes toward oxidation [26]. To prevent damage to proteins, lipids, DNA, and RNA by oxidative stress, cells activate systems of enzymatic and non-enzymatic defense mechanisms. It has been found that oxidative stress can damage oocytes and may impair their fertility capacity. Moreover, overproduction of ROS has a significant negative impact on the IVF success [27]. Recent studies on the pathological characteristics of endometriosis have found that oxidative stress promotes the implantation of ectopic endometrium, and ectopic endometrium produces ROS, thereby creating a vicious cycle that promotes the spread of endometriosis and enhancing infertility in women [28]. ROS production in the healthy organism is in balance with its antioxidant system, the aim was to study oxidative and antioxidant parameters of subjects undergoing oocyte aspiration.

Midazolam, as a benzodiazepine drug, reduces neuronal excitability by increasing the efficiency of the brain neurotransmitter gamma-aminobutyric acid (GABA) and thereby reducing anxiety and sedation [12]. Our hypothesis was that administration of midazolam would result in lower TOC and increased TAC in patients’ serum compared to the control group. The primary outcome is the effect of midazolam on the stress markers during the procedure. There is a decreasing trend in total plasma peroxides in both groups, but it was not statistically significant. We can conclude that administration of midazolam had no effect on total peroxides level. TAC showed a different pattern. While at the first time point in the control group, no significance was observed between the first and third time point. Analysis of TOC in follicular fluid showed that the midazolam group had lower TOC compared to the control group, though not statistically significant. In the serum of the patients, glutathione was monitored as one of the most important components of the antioxidative system. The control group showed a statistically significant decrease in plasma glutathione levels at the second time point. In contrast, there was no statistically significant decrease in plasma glutathione levels in the midazolam group. The results of the glutathione analysis suggest that the control group had increased glutathione consumption, as a measure of stress compensation. Previous work showed that total glutathione levels in follicular fluid were lower in patients with low fertility rates [29]. The work also showed that lipid peroxidase and TAC levels in the follicular fluid were positively correlated with pregnancy rates [30]. Catalase, an important antioxidative enzyme, decreased statistically significantly at the third time point in both groups. However, by comparison, no statistically significant difference was found between the groups.

Data suggest that the immune status contributes greatly to fertility and that, in IVF, follicular fluid immune status contributes significantly to the success of the procedure [31]. Therefore, IL1β was used as a marker of immunological activity in the patients’ follicular fluid. IL1β is an important mediator of the inflammatory response. It primarily promotes the proliferation and differentiation of inflammatory cells and is thought to influence hypersensitivity in the inflammatory response [31]. There is an immune theory that ovulation is actually an immune response, and IL1β has been shown to participate in ovulation induction by facilitating follicular rupture [32]. Lower ILβ levels have been associated with infertility, which could be further related to follicular damage [33]. An analysis of the obtained results of the levels of IL1β in follicular fluid of the patients showed that the midazolam group had higher IL1β levels compared to the control group, although not statistically significant.

The results showed a consistent trend of stress markers reduction in serum, whole blood, and follicular fluid, but are not equivocal and do not confirm either positive or negative effects of midazolam at the biochemical level or at the level of a positive outcome of the IVF procedure. 

Our study is a pilot study conducted on a small number of patients, with an even smaller number of positive outcomes. We aimed here to compare the standard treatment with a novel approach based on a possible additive effect of midazolam premedication. In addition, due to a limited number of patients, it was difficult to confirm with certainty the statistical trends observed. This study points to the need to investigate the effects of axiolysis during IVF on the outcome of this procedure. This is of importance, especially since patients may use anxiolytics either by themselves or by advice of their MD.

## Figures and Tables

**Table 1:** Patients’ hormonal status.

Parameter	Control Group	Midazolam Group	χ2, *p*
Estradiol	H = 3 N = 23 L = 1	H = 5 N = 23 L = 3	1.230; 0.541
Progesterone	H = 0 N = 10 L = 9	H = 2 N = 10 L = 8	2.035; 0.362
Testosterone	H = 0 N = 21 L = 0	H = 1 N = 26 L = 0	0.794; 0.891
Dehidroxyepiandrostendione-sulfate	H = 1 N = 16 L = 0	H = 2 N = 17 L = 0	0.253; 0.503
thyrotropin	H = 0 N = 24 L = 0	H = 0 N = 22 L = 0	-
anti-Müllerian hormone	H = 0 N = 23 L = 0	H = 0 N = 26 L = 0	-
follicle-stimulating hormone	H = 0 N = 24 L = 0	H = 0 N = 26 L = 0	-
luteinizing hormone	H = 4 N = 21 L = 0	H = 0 N = 27 L = 0	4.680; 0.030
Prolactin	H = 3 N = 16 L = 2	H = 5 N = 17 L = 2	0.332; 0.847
BMI	H = 4 N = 23 L = 2	H = 9 N = 21 L = 1	2.281, 0.319

H: number of patients with values above the normal range; N: number of patients with values within the normal range; L: number of patients with values bellow the normal range.

**Table 2:** Hormonal status of pregnant patients.

Parameter for	Control Group N = 8	Midazolam Group N = 3	χ2, *p*
Estradiol	H = 1 N = 4 L = 0	H = 0 N = 3 L = 0	0.686; 0.408
Progesterone	H = 0 N = 1 L = 1	H = 0 N = 2 L = 1	-
Testosterone	H = 0 N = 3 L = 0	H = 0 N = 2 L = 0	-
Dehidroxyepiandrostendione-sulfate	H = 1 N = 2 L = 0	H = 0 N = 2 L = 0	0.833; 0.361
thyrotropin	H = 0 N = 4 L = 0	H = 0 N = 3 L = 0	-
anti-Müllerian hormone	H = 3 N = 1 L = 0	H = 1 N = 1 L = 0	0.375; 0.540
follicle-stimulating hormone	H = 0 N = 5 L = 0	H = 0 N = 3 L = 0	-
luteinizing hormone	H = 1 N = 4 L = 0	H = 0 N = 3 L = 0	0.686; 0.408
Prolactin	H = 1 N = 1 L = 0	H = 0 N = 3 L = 0	1.875; 0.171
BMI	H = 2 N = 4 L = 2	H = 1 N = 2 L = 0	0.000; >0.999

H: number of patients with values above the normal range; N: number of patients with values within the normal range; L: number of patients with values bellow the normal range.

## Data Availability

The data presented in this study are available on request from the corresponding author. The data are not publicly available to due to privacy reasons.
